# Age and fasting blood sugar levels are associated factors for mindful eating among Type 2 diabetes mellitus patients during COVID-19 pandemic confinement

**DOI:** 10.1371/journal.pone.0274327

**Published:** 2022-09-23

**Authors:** Nurul Hayati Chamhuri, Noorlaili Mohd Tohit, Amirah Azzeri, Norshamliza Chamhuri, Siti Rohani M. Alias

**Affiliations:** 1 Family Medicine Unit, Department of Primary Health Care, Faculty of Medicine and Health Sciences, Universiti Sains Islam Malaysia (USIM), Nilai, Negeri Sembilan, Malaysia; 2 Department of Family Medicine, Faculty of Medicine, Universiti Kebangsaan Malaysia (UKM), Cheras, Kuala Lumpur, Malaysia; 3 Public Health Unit, Department of Primary Health Care, Faculty of Medicine and Health Sciences, Universiti Sains Islam Malaysia (USIM), Nilai, Negeri Sembilan, Malaysia; 4 Center for Sustainable and Inclusive Development Studies, Faculty of Economics and Management, Universiti Kebangsaan Malaysia, Bangi, Selangor, Malaysia; 5 Kajang Health Clinic, Malaysia Ministry of Health, Selangor, Malaysia; Ehime University Graduate School of Medicine, JAPAN

## Abstract

The COVID-19 pandemic has impacted the eating behaviours of many people, especially Type 2 Diabetes Mellitus (T2DM) patients. This study aimed to determine the level of mindful eating and its associated factors among T2DM patients at a primary care clinic near Kuala Lumpur. A cross-sectional study was conducted from 18th December 2020 to 5th March 2021 during the movement control order in Malaysia. Respondents were recruited using systematic random sampling via an electronic appointment system. They completed a questionnaire consisting of sociodemographic, clinical profiles, and a Malay-translated Mindful Eating Questionnaire (MEQ-M). Their blood pressure and body mass index were taken during the appointment day while the remaining clinical profiles such as fasting blood sugar (FBS) were obtained from the medical record. Two hundred respondents were recruited with a mean (SD) age of 57.0 (10.90) years. More than half of them were female (54%). Two-thirds of them had uncontrolled diabetes based on elevated FBS of >7 mmol/L (61.5%) and glycated haemoglobin (HbA1c) of >7% (67%), respectively. The mean (SD) score for mindful eating was 2.9 (0.25). Multiple logistic regression revealed that older respondents had a higher level of mindful eating [(AOR = 1.05, *p*-value 0.01, 95% CI = 1.01–1.09)]. In addition, elevated FBS level was also associated with a greater level of mindful eating [(AOR = 2.55, *p*-value 0.01, 95% CI = 1.28–5.07)]. Therefore, healthcare providers should promote mindful eating during the consultation, especially among younger patients. Blood glucose monitoring is also recommended to instil awareness of the importance of healthy eating habits.

## Introduction

The first COVID-19 case was reported in Malaysia on 25th January 2019, followed by an upsurge of cases worldwide since February 2020 [1]. Following the World Health Organization’s (WHO) declaration of COVID-19 as a global pandemic in early March 2020, numerous countries have taken rapid and extensive measures to contain the spread of the virus. In Malaysia, movement control orders with strict standard operation procedures were enforced to control the transmission. Physical confinement measures that were implemented included the closure of wet and night markets, reduction of dining-in capacity at eateries, and limitation of the number of people in retail shops or supermarkets. In addition, essential services like health clinics were permitted to open but with a strict limitation on the patients that can be seen per day. The restrictions on movements and premise operations meant that many people resorted to eating at home by cooking or ordering in via food delivery services. This subsequently modified the eating behaviours of many people, including those with comorbidities such as Type 2 Diabetes Mellitus (T2DM).

T2DM is a metabolic disease characterised by hyperglycaemia and insulin resistance. The prevalence of T2DM among adults above 18 years old in Malaysia has alarmingly risen from 17.5% in 2015 to 18.3% in 2019 [2]. T2DM is associated with an increased risk of severe morbidity and mortality from COVID-19 [3]. In a systematic review of changes in eating behaviours during the pandemic, the results showed a higher tendency to resort to unhealthy eating behaviours, especially increased frequency in consuming snack meals and comfort food such as sweets or starchy food [4]. This is also consistent with a study among T2DM patients in Spain that reported an increased intake of snacks and sugary food compared to vegetable consumption. Furthermore, the frequency of snack consumption is associated with food cravings [5]. Although there are more imminent risks for T2DM patients with COVID-19 infection, the increased psychological stress caused by the global epidemic may leave a prolonged effect on the eating behaviours of diabetic patients [6, 7]. Therefore, it is important to determine the practice and potential benefits of mindful eating during this period.

Mindful eating is defined as the self-regulation of attention towards food and eating experience in a non-judgmental manner [8]. Mindful eating refers to eating consciousness, especially in the sense of noticing the flavours and textures of food with all the senses. It also entails the awareness of the eating pattern and regulation of external triggers to eating [9]. Mindful eating cultivates the habit of listening to internal body cues so that people can control their food intake by responding appropriately to hunger and fullness. It also improves their awareness of external food stimuli so that they can respond in a more calm and contained manner. Mindfulness-based interventions such as mindfulness-based stress reduction (MBSR), mindfulness cognitive therapy (MBCT), and mindfulness eating intervention (MB-EAT) are effective in reducing anxiety, depression, and diabetic distress amongst T2DM patients [10–13]. However, the effect of mindfulness-based interventions on glycated haemoglobin (HbA1c), a test that measures average plasma glucose level over three months, remains inconclusive in the literature [8].

On the other hand, a randomised controlled trial (RCT) that compared MB-EAT with standard diabetes self-management (DSMA) demonstrated that depressive symptoms, self-efficacy of eating, and HbA1c levels improved significantly from baseline levels in both groups [14]. However, the DMSA group showed a significantly higher level of knowledge and self-efficacy in eating while the MB-EAT group recorded a higher mindfulness level. Therefore, as both treatments were equally effective, mindful eating training could improve diet control among T2DM patients [14].

To date, there is a lack of studies assessing mindful eating levels during the COVID-19 pandemic in Malaysia. This study aimed to determine the level of mindful eating among T2DM patients at a primary care clinic using the Malay-translated Mindful Eating Questionnaire (MEQ-M) and to identify the sociodemographic and clinical factors associated with mindful eating. The findings can improve the awareness of mindful eating among healthcare practitioners for them to promote it to T2DM patients.

## Methodology

### Study design

This cross-sectional study was conducted from 18th December 2020 to 5th March 2021 at a primary care clinic near Kuala Lumpur. Using the single mean formula, the sample size calculation based on a 95% confidence interval, 5% absolute precision, and a standard deviation of 0.33 obtained from a previous study of mindful eating among the general population showed that 167 respondents were required [15]. The sample size was further inflated by 20% to address potential non-response, giving a total sample size was 200. The respondents were selected using systematic random sampling from the sampling frame of the T2DM patients’ appointment list in the electronic clinic appointment system (Tele primary care system), with a sampling interval of five patients.

Malaysian citizens aged 18 years and above who attended diabetic clinic follow-up and were Malay-literate were invited to participate. Those with type 1 diabetes mellitus and gestational diabetes were excluded. This study received ethical approval from the National Medical Research Ethical Committee (registered as ID 20-1084-54436). Written informed consent was obtained from the respondents before they were asked to complete a set of questionnaires. The whole process took about 15 minutes. An on-site researcher was available if the respondents needed further explanation and clarification.

### Study instrument

Respondents were required to fill out a questionnaire consisting of three sections. Section A comprised sociodemographic characteristics including age, gender, ethnicity, educational level, employment status, and total household income. Section B documented clinical profiles such as duration, treatment, family history of T2DM, body mass index (BMI), fasting blood sugar (FBS), HbA1c levels, blood pressure, and other co-morbidities. BMI and blood pressure were recorded on the appointment day using a standardised stadiometer with a weighing scale and an electronic blood pressure machine. The BMI cut-off point was based on the Malaysian guideline [16]. The remaining clinical profiles were obtained from the medical record. The latest FBS and HbA1c levels within the last six months were extracted.

Section C was a validated local adaptation of the MEQ-M questionnaire with permission from the authors [17, 18]. The MEQ-M showed reasonable internal consistency reliability in a previous study (Cronbach’s alpha, α = 0.64) [18]. The test-retest reliability coefficient was 0.295, indicating a fair agreement between the scores [18]. The questionnaire consists of 28 questions within five domains that are arranged at random and scored on a Likert scale of 1 to 4 (4 = usually/ always, 3 = sometimes, 2 = often, and 1 = never/rarely). The five subdomains are awareness, distraction, disinhibition, emotional, and external cues. The minimum and maximum scores of the MEQ-M are 24 and 112 respectively, with a mean score ranging from 1 to 4. A higher level of mean score indicates a higher level of mindful eating.

### Data analysis

Data were analysed using SPSS (Version 26). A descriptive analysis was conducted to determine the levels of mindful eating and its subdomains, as well as the sociodemographic and clinical profiles of the respondents. The results were presented as frequencies and percentages for categorical variables. For numerical variables, normally distributed data were expressed as mean (SD) while non-normally distributed data were expressed as median (IQR). Simple and multiple logistic regression analyses were used to determine independent predictors of mindful eating practice. The regression model fits reasonably well. There was no multicollinearity and interaction between all the independent variables tested. Statistical significance was taken as p-value < 0.05 [19].

## Results

Table 1 shows the respondents’ sociodemographic characteristics. More than half of the respondents were females (54%). The mean (SD) of age was 57.0 (10.90) years. Overall, the majority of the respondent were older-age Malay females from lower education and socioeconomic background.

**Table 1:** Sociodemographic characteristics of the study respondents (N = 200).

Variable	Results
**Age (in years)**, mean (SD)	57.0 (10.90)
**Gender**, n (%)	
Female	108 (54.0)
Male	92 (46.0)
**Ethnicity,** n (%)	
Malay	122 (61.0)
Indian	51 (25.5)
Chinese	23 (11.5)
Others	4 (2.0)
**Education level**^1^, n (%)	
No formal education	10 (5.0)
Lower education (primary & secondary education)	151 (75.5)
Higher education (tertiary education)	39 (19.5)
**Employment status,** n (%)	
Retired or non-employed	126 (63.0)
Employed	74 (37.0)
**Household income**^2^, n (%)	
≤ RM 4,849	155 (80.7)
RM4,850- RM 10, 959	27 (14.1)
> RM 10, 960	10 (5.2)

1. According to the 2013 Malaysian Education Statistics, education levels can be divided into lower or higher education levels. Lower education level includes pre-school to secondary education while higher education includes certificate, diploma, undergraduate, and postgraduate programmes.

2. Household income was defined based on the Household Income and Basic Amenities Report 2019, Department of Statistics, Malaysia.

Table 2 shows the respondents’ clinical profiles. Most of the respondents have been diagnosed with T2DM for more than five years (52.5%). Although two-thirds of respondents had uncontrolled T2DM, only one-third of them were on insulin therapy (31%). In addition, most of them were overweight or obese (88.0%).

**Table 2:** Clinical profile of the study respondents (N = 200).

Variable	Frequency, n (%)	Mean (SD)/ Median (IQR) *
**Duration of T2DM** (in years)		6.0 (10.00) *
≤ 5 years	95 (47.5)	
> 5 years	105 (52.5)	
**Treatment of T2DM**		
Single oral antidiabetics agent (OADs)	52 (26.4)	
Two or more OADs	81 (41.1)	
OAD+ insulin	61 (31.0)	
Others: diet	3 (1.5)	
**Family history of diabetes**		
Yes	144 (72.0)	
No	56 (28.0)	
**BMI** (kg/m^2^)		29.3(5.73)
**BMI categories**		
Underweight/normal (BMI < 22.9)	24 (12.0)	
Overweight (BMI 23–27.4)	56 (28.0)	
Obese (BMI ≥ 27.5)	120 (60.0)	
**FBS level**^**1**^ (mmol/L)		7.7(3.70) *
≤ 7 mmol/L	77 (38.5)	
> 7 mmol/L	123 (61.5)	
**HbA**_**1**_**c level** (%)		8.0 (2.00)
**HbA**_**1**_ **c grouping** ^**2**^		
Uncontrolled (≥ 7%)	134 (67.0)	
Controlled (< 7%)	66 (33.0)	
**Blood pressure**(mmHg)		
Systolic blood pressure (SBP)		138.2 (15.60)
Diastolic blood pressure (DBP)		80.1 (9.17)
**Co-morbidities**		
Hypertension	159 (79.5)	
Dyslipidaemia	133 (66.8)	
Cardiovascular disease	17 (8.5)	
Cerebrovascular disease	8 (4.0)	
Chronic kidney disease (eGFR <60 mL/min)	6 (3.0)	

*All continuous data were normally distributed except the duration of T2DM and FBS level.

1. FBS of ≤ 7 mmol/L is considered normal while an FBS of > 7 mmol/L is abnormally deranged (American Diabetes Association, 2021).

2. HbA1c of < 7.0% is considered good glycaemic control while HbA1c ≥ 7.0% is deemed to be poor glycaemic control (American Diabetes Association, 2021).

Next, the mean (SD) score for mindful eating was 2.9 (0.25) (Table 3). The emotional subdomain recorded the highest score, which was 3.5 (0.56). In contrast, the subdomain of external cues demonstrated the lowest score with 1.7 (0.51). In other words, the respondents were more likely to eat in response to food and environmental cues such as food advertisements or eating at a social function because the food was present. In contrast, they were likely to be not eating due to negative emotional reactions.

**Table 3:** The Mean Score of Mindful Eating and its’ subdomains (N = 200).

Domain	Mean (SD)
Mindful eating	2.9 (0.25)
**Subdomains**	
Awareness	2.6 (0.59)
Distraction	3.5 (0.57)
Disinhibition	3.2 (0.50)
Emotional	3.5 (0.56)
External cues	1.7 (0.51)

*All continuous data were normally distributed.

Similar to Chung et al., an arbitrary cut-off point on the level of mindful eating was made using the mean score to facilitate the interpretation of the study results [20–22]. Therefore, any scores above 2.90 were considered to be a high level of mindful eating. Simple logistic regression (SLR) and multiple logistic regression (MLR) were performed to identify the predictors of mindful eating (Table 4). All independent variables with a p-value of < 0.25 were further analysed using the ‘ENTER’ method in MLR. Age and FBS levels were significantly associated with a high level of mindful eating. As age increased, the odds of practising a high level of mindful eating also increased (AOR = 1.05, 95% CI = 1.01–1.09). A relatively small confidence interval showed a high precision rate of the results. Those with elevated FBS levels were 2.55 times more likely to have a high level of mindful eating than those with normal FBS levels (AOR = 2.55, 95% CI = 1.28–5.07).

**Table 4:** Association of the level of mindful eating with sociodemographic characteristics and clinical profiles (N = 200).

Variable	Mindful eating level		Crude OR (95% CI)	p-value^a^	β	Adjusted OR (95% CI)	p-value^b^
	High n = 101	Low n = 99					
**Age**, mean (SD)	59 (10.0)	55 (11.0)	1.03 (1.01,1.06)	0.01	0.05	**1.05 (1.01, 1.09)**	**0.01**
**Gender,** n (%)							
Female	59 (54.6)	49 (45.4)	0.84 (0.82, 2.51)	0.20	-0.55	0.57 (0.30,1.1)	0.90
Male	42 (45.7)	50 (54.3)	ref			ref	
**Ethnicity,** n (%)							
Malay	63 (51.6)	59 (48.4)	ref				
Non-Malay	38 (48.7)	40 (51.3)	1.07 (0.50, 1.57)	0.69			
**Education level,** n (%)							
No formal education	3 (30.0)	7 (70.0)	ref			ref	
Lower education *(primary & secondary)*	79 (52.3)	72 (47.7)	2.56 (0.64, 10.27)	0.19	1.36	3.90(0.83,18.46)	0.09
Higher education *(tertiary)*	19 (48.7)	20 (51.3)	2.21 (0.50,9.85)	0.30	1.57	4.79(0.88,26.17)	0.07
**Employment status,** n (%)							
Retired/Non-employed	67 (53.2)	59 (46.8)	ref				
Employed	34 (45.9)	40 (54.1)	0.75 (0.42,1.33)	0.32			
**Household income,**							
n (%)							
≤ RM 4,849	78 (50.3)	77 (49.7)	ref				
RM 4,850-RM10,959	14 (51.9)	13 (48.1)	1.06 (0.47, 2.41)	0.88			
> RM 10,960	5 (50.0)	5 (50.0)	0.99 (0.28, 3.55)	0.98			
**Duration of T2DM**, in years							
≤ 5 years	45 (47.4)	50 (52.6)	ref				
> 5 years	56 (53.3)	49 (46.7)	1.27 (0.73, 2.21)	0.40			
**Treatment of T2DM**							
Diet/OADs	70 (51.5)	66 (48.5)	ref				
Insulin	29 (47.5)	32 (52.5)	0.85 (0.47,1.56)	0.61			
**Family history of T2DM**							
Yes	72 (50.0)	72 (50.0)	ref				
No	29 (51.8)	27 (48.2)	1.07 (0.58, 1.99)	0.82			
**BMI,** mean (SD)	28.4 (4.8)	30.3 (6.4)	0.94 (0.89,0.99)	0.03	-0.04	0.96 (0.91, 1.02)	0.22
**FBS**, n (%)							
≤ 7 mmol/L	33 (42.9)	44 (57.1)	ref			ref	
> 7mmol/L	68 (55.3)	55 (44.7)	1.65 (0.93, 2.93)	0.09	0.94	**2.55 (1.28, 5.07)**	**0.01**
**HbA1c level**, mean (SD)	8.00 (2.0)	8.05 (2.1)	0.99 (0.86,1.14)	0.88			
**Systolic blood pressure,** mean (SD)	137 (15.0)	140 (17.0)	0.98 (0.97,1.00)	0.19	-0.02	0.98 (0.96,1.00)	0.06
**Diastolic blood pressure,** mean (SD)	79 (9.0)	81 (9)	0.98 (0.95,1.00)	0.15	0.01	1.01 (0.97, 1.06)	0.53
**Co-morbidities**							
Hypertension	79 (49.7)	80 (50.3)	0.85 (0.43,1.69)	0.65			
Dyslipidaemia	72 (54.1)	61 (45.9)	1.60 (0.88,2.90)	0.12	0.46	1.59	0.16
Cardiovascular disease	9 (52.9)	8 (47.1)	1.11 (0.41, 3.01)	0.83		(0.83,3.05)	
Cerebrovascular disease	5 (62.5)	3 (37.5)	1.67 (0.39,7.17)	0.50			
Chronic kidney disease	6 (100.0)	0 (0.0)	UTC	0.99			

Hosmer-Lameshow test (p = 0.98), classification table (overall correctly classified percentage = 60.8%), and area under the ROC curve (69.8%) were applied to check the model fit. **BOLD: significant results;** UTC: unable to compute. a: simple logistic regression, b: multiple logistic regression.

## Discussion and conclusion

This study estimated the mean scores of mindful eating among T2DM patients during the COVID-19 pandemic. From the sociodemographic aspect, older age was an independent factor associated with high levels of mindful eating. This is consistent with previous literature highlighting a significant association between age and mindful eating practice [23, 24]. However, since most of the study respondents in this study were older adults, the results might not be generalisable to other age groups. Gender, ethnicity, and education level were not associated with the level of mindful eating, a similar finding in other literature [17, 18, 24]. Interestingly, this study also found that employment status and socioeconomic background were not associated with a mindful eating level. However, theoretically, people from a low socioeconomic background would experience a higher level of psychosocial stressors and they may benefit the most from mindful eating training [25].

To the best of our knowledge, there is a lack of studies on mindful eating among T2DM patients, especially in the local setting. Hence, this study provided a fundamental concept of mindful eating among T2DM patients, especially in the region of Southeast Asia and Malaysia. Elevated FBS level was an independent factor of high levels of mindful eating. As such, FBS monitoring is an important measure to instil awareness of mindful eating in the efforts to improve the eating behaviour of T2DM patients. Studies revealed that T2DM patients were most familiar with their FBS levels among all the blood parameters [26, 27]. In addition, having a good knowledge of blood sugar levels was a predictor of good glycaemic control and better self-care [28, 29]. Other clinical profiles such as duration and treatment of diabetes, HbA1c levels, BMI, blood pressure, and comorbidities were not associated with mindful eating. This may suggest that the relationship between these variables, especially HbA1c and mindful eating was not straightforward.

Even though a few studies have reported that a higher BMI was associated with lower mindful eating levels, no significant association was observed in this study [24, 30–32]. The difference could be attributed to different study populations in which this study focused more on T2DM patients who were also obese while previous studies were conducted among obese populations in communities [24, 30–32] However, further in-depth studies are needed to examine the effects of mindful eating training among T2DM patients with obesity, regardless of whether the underpinning issue of their eating behaviours is the same as other obese populations.

The mean (SD) for mindful eating levels among T2DM patients in this study was 2.9 (0.25). Studies in various populations in America, Turkey, and Australia found that the mean (SD) scores of mindful eating ranged from 2.9 (0.32) to 3.5 (0.45) [17, 23, 24, 30] while developing countries such as Malaysia, Iran, and Mexico recorded slightly lower mean (SD) scores of mindful eating, that were between 2.6 (0.25) and 2.8 (0.48) [18, 31–33]. With regard to other medical conditions, a study among American breast cancer survivors showed that the baseline mean (SD) score of mindful eating of 2.9 (0.40) increased significantly after 12 weeks of mindful eating intervention [20]. In addition, two studies have detected a relatively higher level of mindful eating among women with gestational diabetes mellitus [23, 34]. On the other hand, the obese population generally practised a lower level of mindful eating, as shown by several studies [24, 30–32].

During the COVID-19 confinement, several studies on eating behaviour highlighted a greater frequency of overeating and snacking among the general population, two habits that were consistently observed among T2DM patients [35–37]. In comparison, areas less affected by COVID-19 in the earlier stages, such as Hong Kong, showed a significantly higher level of healthy eating behaviour [38]. Two significant factors affecting eating behaviour during this period included a higher stockpile of food at home and emotional eating due to the stress from the lockdown [35]. This study showed that the higher food supplies at home significantly affected eating behaviour. The study population could be more prone to eating due to external cues when food or snacks were easily available at home, apart from indulging themselves in emotional eating. Therefore, should there be a pandemic or similar lockdowns in the future, the government and stakeholders should be aware of this to ensure a good supply of healthy food.

In addition, eating behaviours among T2DM patients also include restrictive eating patterns, restrained eating, overeating, under-reporting of eating habits, and non-adherence to diet control [39]. According to Schachter’s externality theory of eating behaviour, those prone to eating in response to external cues are often obese and on a prolonged restrained diet, subsequently leading to a higher energy intake [40, 41]. More randomised clinical trials on mindful eating are needed to examine the long-term effect of mindful eating training to help T2DM patients in complying better with a carbohydrate restriction diet [42].

Furthermore, it is vital for the government and stakeholders to incorporate mindful eating training as part of diabetes self-management to curb overeating and habitual eating [6, 42]. The module and training should include mindful eating exercise, relaxation techniques or meditation, bodily awareness of hunger, and satiety cues [6]. It should be developed by dieticians in collaboration with clinical psychologists. Clinicians must recognise potential dysregulation of eating habits among T2DM patients to promote individual awareness of mindful eating. Such promotion can be made widely through a mobile application that teaches patients mindful eating techniques [42].

There are several limitations to this research. The MEQ-M is a self-reported questionnaire and thus subjected to possible self-reporting bias. For instance, the respondents might have reported proper practice of eating as socially expected instead of their actual eating habits. Furthermore, caution is needed when interpreting our results due to the single-centre setting. Our findings would only be generalisable to the local population who attended primary care clinics. We recommended further studies on mindful eating with a bigger sample size among the multi-ethnic populations in both rural and urban areas. It is also wise to repeat the observation post-COVID-19 to determine if there is any difference in results.
